# Cytokine pre-activation of cryopreserved xenogeneic-free human mesenchymal stromal cells enhances resolution and repair following ventilator-induced lung injury potentially via a KGF-dependent mechanism

**DOI:** 10.1186/s40635-020-0295-5

**Published:** 2020-02-05

**Authors:** Shahd Horie, Sean Gaynard, Mary Murphy, Frank Barry, Michael Scully, Daniel O’Toole, John G. Laffey

**Affiliations:** 1 Anaesthesia, School of Medicine, National University of Ireland, Galway, Ireland; 2 Regenerative Medicine Institute (REMEDI) at CÚRAM Centre for Research in Medical Devices, National University of Ireland Galway, Galway, Ireland; 3 Medicine, School of Medicine, National University of Ireland, Galway, Ireland; 4 Department of Anaesthesia, Galway University Hospitals, Saolta University Health Group, Galway, Ireland

**Keywords:** Acute respiratory distress syndrome, Ventilation-induced lung injury, Injury resolution and repair, Mesenchymal stem/stromal cells, Cryopreservation, Cell activation

## Abstract

**Background:**

Human mesenchymal stem/stromal cells (hMSCs) represent a promising therapeutic strategy for ventilator-induced lung injury (VILI) and acute respiratory distress syndrome. Translational challenges include restoring hMSC efficacy following cryopreservation, developing effective xenogeneic-free (XF) hMSCs and establishing true therapeutic potential at a clinically relevant time point of administration. We wished to determine whether cytokine pre-activation of cryopreserved, bone marrow-derived XF-hMSCs would enhance their capacity to facilitate injury resolution following VILI and elucidate mechanisms of action.

**Methods:**

Initially, in vitro studies examined the potential for the secretome from cytokine pre-activated XF-hMSCs to attenuate pulmonary epithelial injury induced by cyclic mechanical stretch. Later, anaesthetised rats underwent VILI and, 6 h following injury, were randomized to receive 1 × 10^7^ XF-hMSC/kg that were (i) naive fresh, (ii) naive cryopreserved, (iii) cytokine pre-activated fresh or (iv) cytokine pre-activated cryopreserved, while control animals received (v) vehicle. The extent of injury resolution was measured at 24 h after injury. Finally, the role of keratinocyte growth factor (KGF) in mediating the effect of pre-activated XF-hMSCs was determined in a pulmonary epithelial wound repair model.

**Results:**

Pre-activation enhanced the capacity of the XF-hMSC secretome to decrease stretch-induced pulmonary epithelial inflammation and injury. Both pre-activated fresh and cryopreserved XF-hMSCs enhanced resolution of injury following VILI, restoring oxygenation, improving lung compliance, reducing lung leak and improving resolution of lung structural injury. Finally, the secretome of pre-activated XF-hMSCs enhanced epithelial wound repair, in part via a KGF-dependent mechanism.

**Conclusions:**

Cytokine pre-activation enhanced the capacity of cryopreserved, XF-hMSCs to promote injury resolution following VILI, potentially via a KGF-dependent mechanism.

## Background

Mechanical ventilation is a potentially life-saving manoeuvre in patients with acute respiratory distress syndrome (ARDS) [1, 2], but it can also exacerbate lung damage—this is termed ventilation-induced lung injury (VILI) [3]. Mesenchymal stem/stromal cells (MSCs) demonstrate beneficial effects in diverse pre-clinical lung injury models including pulmonary [4–6] and abdominal sepsis [7–9], bleomycin-induced acute lung injury [10] and fibrosis [11, 12]. Human-derived MSCs (hMSCs) enhance injury resolution following established VILI [13, 14], enhance recovery of human lungs ex vivo [15] and reduce the severity of *Escherichia coli* pneumonia [16]. Finally, recent phase 1–2 studies suggest that allogeneic bone marrow-derived hMSCs (BM-hMSCs) can be safely administered to patients with moderate to severe ARDS [17, 18].

In regard to clinical translation of hMSC therapies, concern exists that the cryopreservation and thawing processes may reduce the efficacy of hMSCs and its secretome [19]. The use of xenogeneic products, particularly foetal bovine serum (FBS)-based media for passage of MSCs [20], has generated important safety concerns [21], including the risk of virus and prion contamination, and concerns regarding immunogenicity [22–25]. Xenogeneic culture-free (XF) supplements such as the recently patented XF supplement (WO2015121471 A1) have been developed that preserve the differentiation, proliferation and low immunogenicity properties of MSCs [26]. Finally, demonstrating true ‘therapeutic’ potential of MSC therapies, i.e. showing efficacy at later points (delayed administration) in the injury and/or repair process, is necessary to better mimic the clinical scenario.

Cytokine activation of hMSCs may enhance their function by simulating the inflammatory/injury microenvironment [27–29], potentially minimizing any impact of cryopreservation, XF culture conditions or loss of therapeutic efficacy with delayed delivery following disease onset. We wished to test the hypothesis that pre-activation of cryopreserved, XF-hMSCs would enhance their efficacy after delayed administration in a relevant preclinical model of VILI injury and repair and to investigate the mechanisms underlying these effects. In vitro studies examined the potential for naive and cytokine pre-activated XF-hMSC-conditioned medium (CM) to attenuate pulmonary epithelial stretch-induced injury. In vivo experiments examined the potential for cytokine pre-activation to enhance the efficacy of (fresh and cryopreserved) XF-hMSCs to enhance resolution when administered at therapeutically relevant time points following the development of VILI. Subsequent mechanistic experiments examined the potential for the pulmonary epithelial reparative effects of XF-hMSCs to be mediated in part via KGF present in the MSC secretome.

## Materials and methods

### hMSC isolation, culture and expansion

hMSCs were isolated from healthy donor bone marrow as previously described [30] and used at passages 2–3 for all experiments. MSCs were cultured in Alpha Minimum Essential Eagle Medium (MEM-α) with GlutaMAX (GIBCO®) supplemented with 10% FBS, penicillin G (100 U/mL), streptomycin (100 μg/mL) and FGF-1 (10 ng/mL) (PeproTech EC Ltd., London, UK). hMSCs were maintained in 95% humidity, 5% CO_2_ and hypoxia (2% O_2_) at 37 °C; sub-cultured with 0.025% trypsin-0.05 mM EDTA; and cryopreserved in CryoStor cell preservation medium (Sigma-Aldrich) at a density of 5 × 10^6^/mL. XF-hMSCs were isolated as above but expanded using an FBS-free medium containing a patented XF (WO2015121471 A1) growth supplement [26]. Following expansion, hMSCs were pre-activated with cytokine cocktail consisting of interleukin (IL)-1β (10 ng/mL), tumour necrosis factor (TNF)-α (50 ng/mL) and interferon (IFN)-γ (50 ng/mL) for 24 h, and either delivered freshly harvested or cryopreserved and stored for later delivery. IL-8 secretion from naive or pre-activated hMSCs, before and after freezing (24 and 48 h post-cryopreservation), was determined using an IL-8 sandwich ELISA DuoSet kit (R&D Systems Inc., Minneapolis, MN, USA) to confirm the responsive state persisted post-thaw (Additional file 1: Figure S1). For in vivo experiments, cryopreserved XF-hMSCs were stored for up to 2 months and cell viability after thaw was between 95 and 97% as determined by trypan blue exclusion.

### hMSC-conditioned medium

hMSCs were seeded at 1 × 10^4^ cells/cm^2^ in a 175-cm^2^ culture flask and left to reach confluence for 48 h. The cells were then re-fed with complete FBS medium or XF medium with or without cytokine cocktail for 24 h. For naive CM, phosphate-buffered saline (PBS) vehicle was added for 24 h. All cells were washed with PBS three times and re-fed with serum-free medium, to remove pre-activating cytokines. This CM was harvested 24 h later. Serum-free MEM-α medium was used for the control treatment groups in experiments. Multiple donors and multiple batches were used in all experiments.

### Pulmonary epithelial stretch injury model

A549/NF-κB-luc cells were seeded to laminin-coated 6-well BioFlex plates (Flexcell International, Burlington, NC, USA) at 1 × 10^5^ cells/cm^2^ and incubated for 48 h. They were then pre-conditioned in their respective hMSC CM treatment or control (MEM-α medium) conditions for 1 h before they were mounted onto the Flexcell FX-4000 T® Tension Plus® baseplate (Flexcell International) and subjected to 22% equibiaxial stretch at a frequency of 0.1 Hz for 120 h. Non-stretched cells were used as control [31]. Cells and medium were then harvested for analysis. Cells were scraped into 1 mL of PBS, centrifuged at 400×*g* for 5 min and reconstituted in 1 mL of PBS. 50 μL was taken for the viability assay and the remainder then pelleted again for the luciferase assay.

### Injury assessment

IL-8, an NF-κB-dependent cytokine, was measured in the medium using an IL-8 sandwich ELISA DuoSet kit (R&D Systems). Lactate dehydrogenase (LDH) was measured to assess cell membrane integrity using the CytoTox 96 Non-Radioactive Cytotoxicity Assay Kit (Promega Corporation, Fitchburg, WI, USA), as per the manufacturer’s instructions. Cellular NF-κB activity was measured by mixing cell pellets with 50 μL of SolarGlow SuperBright (Molecutools, Dublin, Ireland), agitating for 5 min and assessing luminescence using a VICTOR™ X plate reader (Perkin Elmer, Waltham, MA, USA). An MTT (Sigma-Aldrich) assay was performed to assess cell viability as previously described [32].

### Ventilator-induced lung injury

As previously described [33–35], rats were anaesthetised with isoflurane and intravenous access was obtained via the tail vein. Laryngoscopy was performed, and a 14-G catheter (BD Insyte®; Becton Dickinson Ltd., Oxford, UK) was used to intubate the animal for ventilation using a small animal ventilator (CWE SAR 830 AP; CWE Inc., Ardmore, PA, USA). Anaesthesia was maintained with repeated boli of Alfaxan® (Alfaxadone 0.9% (w/v) and alfadolone acetate 0.3% (w/v); Vétoquinol S A, Lure Cedex, France) and paralysis with cisatracurium besylate 0.5 mg. kg^−1^ (GlaxoSmithKline, Dublin, Ireland). Following baseline ventilation, static compliance was measured and VILI was induced using the following ventilator settings: Fi_O2_ of 0.3, *P*_insp_ 35 cmH_2_O, respiratory rate 18 min^−1^ and PEEP 0 cmH_2_O. Following the development of severe VILI, as evidenced by a 50% decrease in respiratory static compliance, injurious ventilation was discontinued, and the animals allowed to recover from anaesthesia [34].

### Experimental design

Six hours following cessation of injurious ventilation, animals were randomized to receive, by intravenous administration, either (i) vehicle (1 mL PBS) or 1 × 10^7^ XF-hMSCs/kg that were (ii) fresh naive hMSCs, (iii) cryopreserved naive hMSCs, (iv) fresh pre-activated hMSCs and (v) cryopreserved pre-activated hMSCs. The extent of inflammation and injury resolution was measured at 24 h (i.e. 18 h post-intervention delivery).

### In vivo assessment of lung injury and recovery

Twenty-four hours post-cessation of injurious ventilation, animals were re-anaesthetised as described above, intravenous access was obtained via tail vein and a tracheostomy tube was inserted [34]. Following the commencement of ventilation, intra-arterial access was gained and anaesthesia was maintained with Saffan® and paralysis with cisatracurium besylate. Arterial blood pressure, airway pressure, lung static compliance and arterial blood gas analyses were performed as previously described [36, 37].

### Ex vivo analyses of lung inflammation and repair

Following exsanguination under anaesthesia, bronchoalveolar lavage (BAL) was collected, and BAL fluid differential leukocyte counts were completed. BAL concentrations of CINC-1, IL-6, IL-10, KGF and PGE_2_ were determined using ELISA (R&D Systems), and BAL protein was also measured (Micro BCA; Pierce, Rockford, IL, USA). The left lung was isolated and fixed, and structural lung damage determined using stereological techniques [38], a quantitative, robust and reproducible approach to histological assessment [39]. All ex vivo analyses were performed by blinded investigators.

### Pulmonary epithelial wound injury

A549/NF-κB-luc cells were seeded at 1 × 10^5^ cells/cm^2^ in a 24-well plate (Sarstedt) and left to reach confluence for 48 h. Single scratch wounds were generated with a 1-mL pipette tip (Sarstedt). The cells were washed with PBS, and their respective treatments were added. Ten per cent serum was also added. Cell treatments were MEM-α medium, naive FBS- or XF-MSC CM, or pre-activated FBS- or XF- MSC CM +/−, a KGF neutralization antibody (0.5 μg/mL) (R&D Systems). Wound restitution was assessed over 48 h using light microscopy imaging.

### Statistical analysis

The distribution of all data was tested for normality using Kolmogorov-Smirnov tests. Data was analysed by one-way or repeated measures ANOVA, with post hoc Student-Newman-Keuls for between-group comparisons, and is presented as mean ± standard deviation. A two-tailed *P* value of < 0.05 was considered statistically significant.

## Results

### In vitro assessments

#### Pulmonary epithelial cell stretch-induced injury

**Fig. 1:**
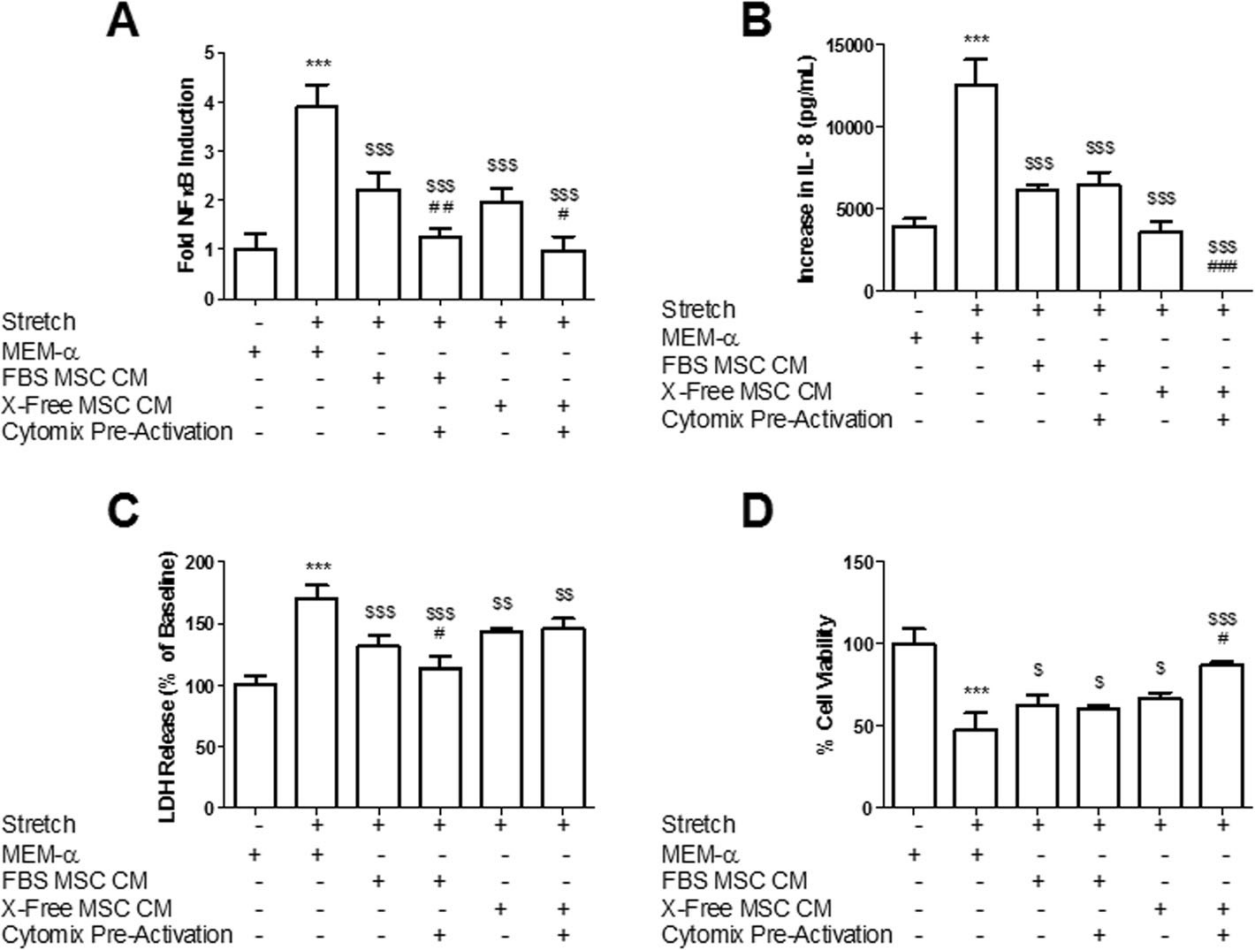
Pre-activation of hMSCs enhances the efficacy of CM in attenuating mechanical stretch injury. Mechanical stretch induction of NF-κB was reduced by FBS- and XF-cultured MSC CM in comparison with the MEM-α stretch control (**a**). Pre-activation of both FBS- and XF-cultured MSCs further enhanced the ability of the MSC CM to attenuate NF-κB when compared to naive MSC CM. Mechanical stretch-induced release of IL-8 (**b**) was ameliorated by both naive CM groups but more significantly so by the pre-activated XF-hMSC CM (**b**). Mechanical stretch-induced LDH release again was attenuated by all the MSC CM groups, but more so in the pre-activated FBS MSC CM group (**c**). The decrease in cell viability, as induced by mechanical stretch, was also abrogated by both naive MSC CM treatments but was more enhanced in the pre-activated XF-hMSC CM group (**d**). ***P < 0.001 versus MEM-α non-stretch control; ^$^, ^$$^ and ^$$$^*P* < 0.05, 0.01 and 0.001, respectively, versus MEM-α stretch; ^#^, ^##^ and ^###^*P* < 0.05, 0.01 and 0.001, respectively, versus respective naive FBS or XF-hMSC CM. MEM-α non-stretch control, *n* = 6; MEM-α stretch control, *n* = 5 or 6; all other groups, *n* = 2 or 3

Cyclic mechanical stretch-induced NF-κB activation was attenuated by both FBS- and XF-cultured hMSC-CM as compared to the control (MEM-α) group (Fig. 1a). Cytokine pre-activation of both FBS- and XF-cultured MSCs enhanced the efficacy of hMSC-CM in attenuating NF-κB activation. Cyclic mechanical stretch-induced IL-8 release was attenuated by FBS- and XF-cultured MSC-CM (Fig. 1b). Cytokine pre-activation of XF-cultured—but not FBS-cultured—MSCs enhanced the efficacy of hMSC-CM in attenuating stretch-induced IL-8 release. Cyclic mechanical stretch caused pulmonary epithelial cell membrane injury, as evidenced by LDH release (Fig. 1c). This pulmonary epithelial injury was attenuated by hMSC-CM from both FBS- and XF-cultured MSCs. Cytokine pre-activation of FBS-cultured—but not XF-cultured—MSCs enhanced the efficacy of hMSC-CM in attenuating epithelial injury (Fig. 1c). Cyclic mechanical stretch decreased pulmonary epithelial cell viability, and this decrement in cell viability was abrogated by hMSC-CM from both FBS- and XF-cultured MSCs (Fig. 1d). Pre-activation of XF-cultured—but not FBS-cultured—MSCs further enhanced the efficacy of its CM in maintaining epithelial cell viability.

### Injury resolution following in vivo ventilation-induced ARDS

#### Recovery of lung function

**Fig. 2:**
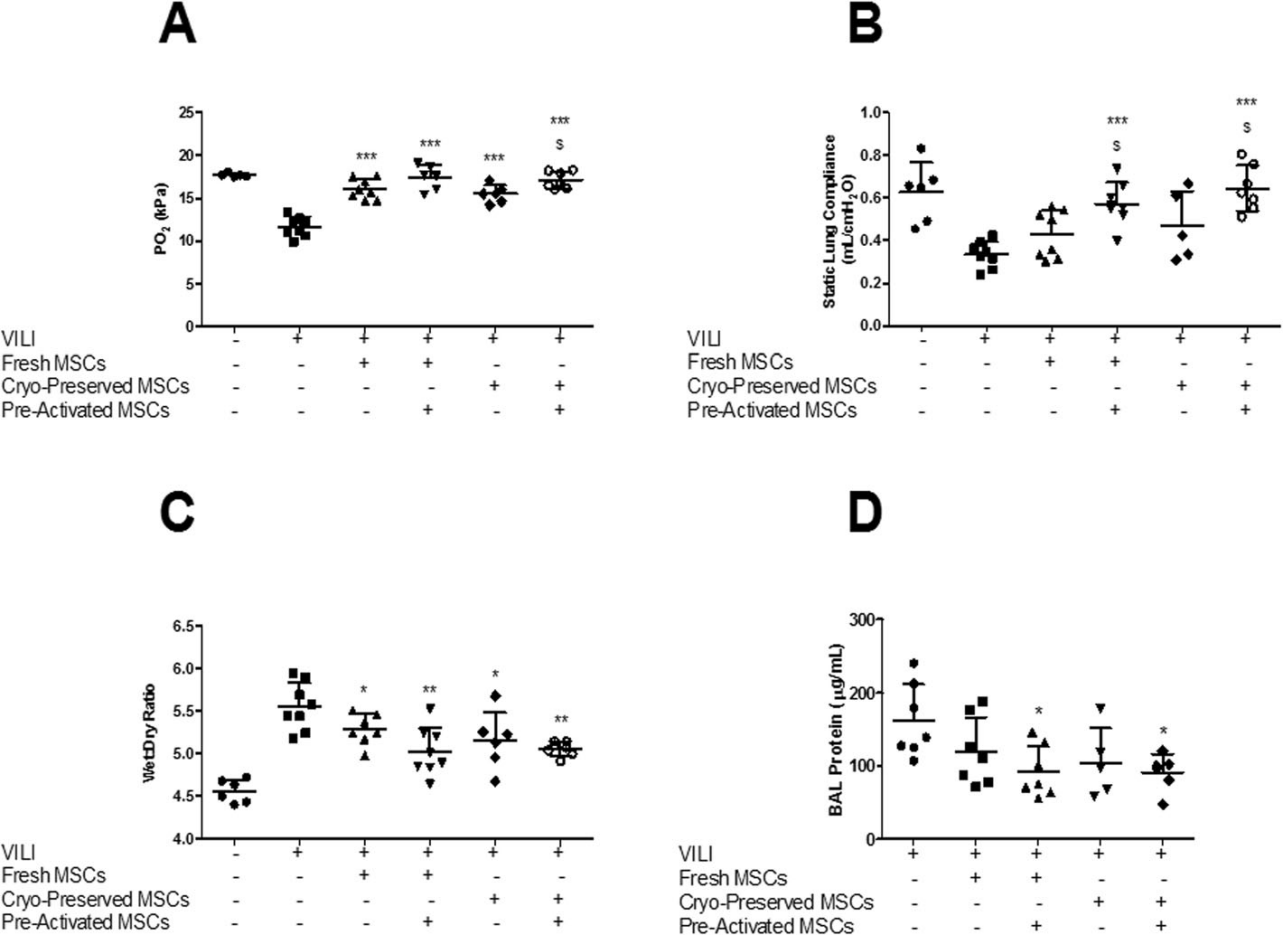
Pre-activated, cryopreserved, XF-hMSCs restore lung function after VILI. All MSC treatments significantly restored arterial PO_2_, with pre-activated cryopreserved cells statistically better than their naive alternatives (**a**). For static lung compliance, only activated fresh and activated frozen delivery showed significant restoration and were statistically different to their naive counterparts (**b**). All treatment groups restored the lung wet to dry ratio (**c**), but only pre-activated MSCs modulated BAL protein content (**d**). *, ** and ***P < 0.05, 0.01 and 0.001, respectively, versus PBS control groups; ^$^*P* < 0.05 versus corresponding naive groups. Sham, *n* = 5–6; PBS control, *n* = 7–8; fresh, *n* = 7–8; fresh pre-activated, *n* = 6–8; cryopreserved, *n* = 5–6; cryopreserved pre-activated, *n* = 6–7

VILI resulted in a significant decrement in oxygenation and lung compliance and a significant increase in lung permeability compared to protective ventilation (Fig. 2). Both fresh and cryopreserved XF-hMSCs restored arterial oxygenation (Fig. 2a) when compared to the vehicle (PBS) control group. Cytokine pre-activation of cryopreserved—but not fresh—XF-hMSCs further enhanced restoration of arterial oxygenation (Fig. 2a). Cytokine pre-activated—but not naive—fresh and cryopreserved XF-hMSCs restored lung compliance (Fig. 2b). Both naive and cytokine pre-activated fresh and cryopreserved XF-hMSCs enhanced resolution of lung oedema (Fig. 2c). Cytokine pre-activated—but not naive—fresh and cryopreserved XF-hMSCs restored alveolar barrier permeability, as evidenced by decreased BAL protein concentrations (Fig. 2d).

#### Modulation of the inflammatory response

**Fig. 3:**
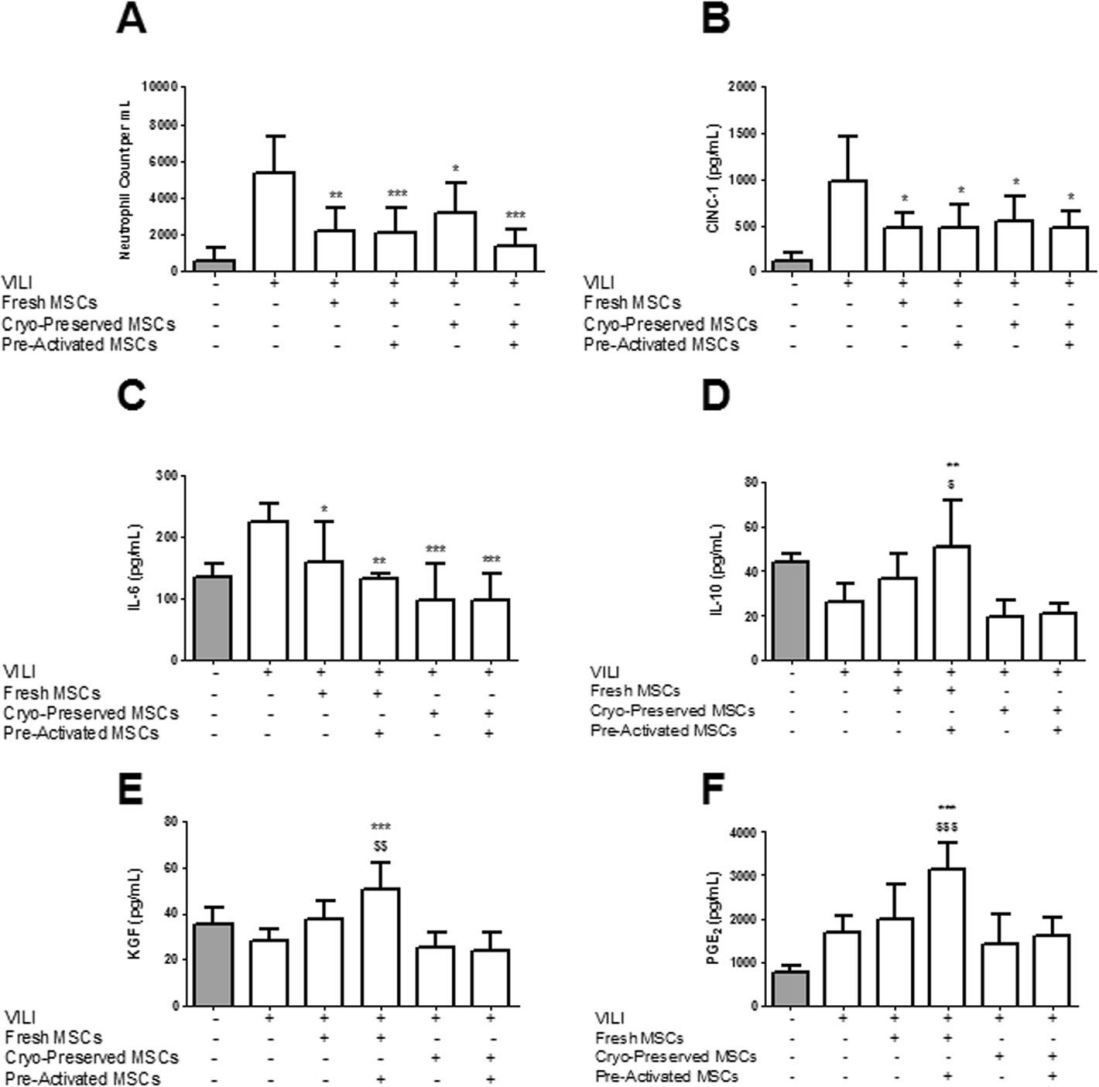
Pre-activated, cryopreserved, XF-hMSCs enhance resolution of alveolar neutrophil infiltration and modulate BAL inflammatory cytokines. All hMSC treatment groups significantly reduced lung neutrophil infiltration (**a**). All treatment groups significantly decreased BAL concentrations of CINC-1 (**b**) and IL-6 (**c**). For IL-10, KGF and PGE_2_, fresh MSC treatment did not restore release (**d**–**f**). However, pre-activated fresh MSCs showed significant recovery of IL-10 (**d**), KGF (**e**) and PGE_2_ (**f**) concentrations. Neither frozen nor activated frozen MSC delivery recovered IL-10, KGF or PGE_2_ release (**d**–**f**). *, ** and ***P < 0.05, 0.01 and 0.001, respectively, versus PBS control; ^$^ and ^$$^*P* < 0.05 and 0.01, respectively, versus naive fresh group. Sham, *n* = 3–6; PBS control, *n* = 6–8; fresh, *n* = 6–8; fresh pre-activated, *n* = 5–8; cryopreserved, *n* = 5–6; cryopreserved pre-activated, *n* = 6–8

Fresh, but not cryopreserved, XF-hMSCs decreased alveolar infiltrating cell counts, while cytokine pre-activation restored the efficacy of cryopreserved hMSCs in reducing alveolar cell counts (Additional file 2: Figure S2). Both fresh and cryopreserved XF-hMSCs decreased alveolar neutrophil counts (Fig. 3a). These effects were not significantly enhanced by cytokine pre-activation of either fresh or cryopreserved hMSCs (Fig. 3a). Fresh and cryopreserved hMSCs—whether naive or pre-activated—significantly attenuated the increase in alveolar CINC-1 (Fig. 3b) and IL-6 (Fig. 3c) concentrations. Pre-activated, but not naive, fresh XF-hMSCs significantly increased alveolar concentrations of anti-inflammatory IL-10 (Fig. 3d), pro-repair KGF (Fig. 3e) and immunomodulatory PGE_2_ (Fig. 3f). Cryopreserved XF-hMSCs, both naive and pre-activated, did not modulate IL-10, KGF or PGE_2_ concentrations (Fig. 3).

#### Restoration of lung structure

**Fig. 4:**
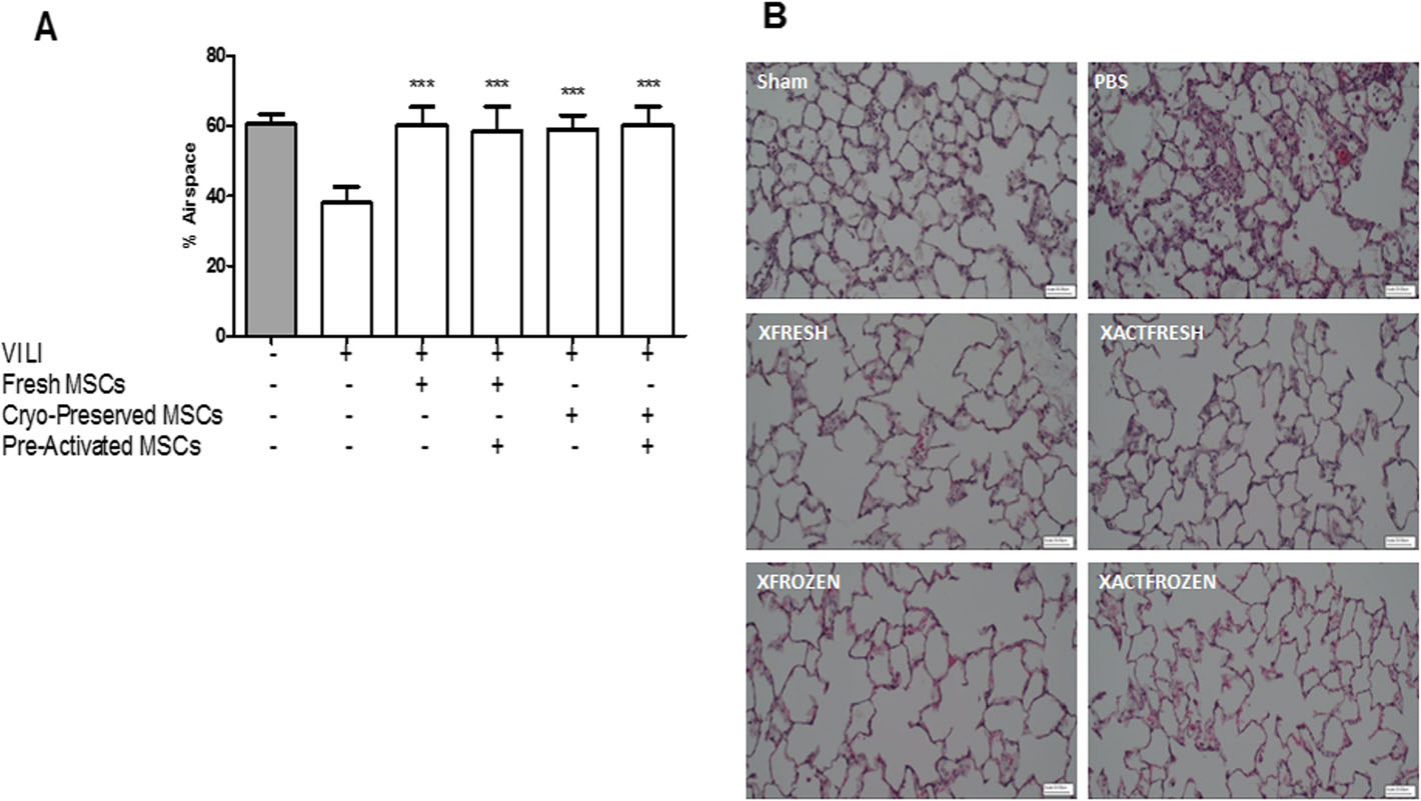
Pre-activated, cryopreserved, XF-hMSCs restore lung structure. All cell treatment groups significantly restored the percentage of alveolar airspace in VILI (**a**). Representative images of lung histology sections are provided (**b**). ***P < 0.001 versus PBS control group. Sham, *n* = 4; PBS control, *n* = 8; fresh, *n* = 8; fresh pre-activated, *n* = 7; cryopreserved, *n* = 6; cryopreserved pre-activated, *n* = 7

Treatment with fresh or cryopreserved hMSCs—both naive or pre-activated—fully restored lung histologic structure post-VILI as assessed by percentage airspace (Fig. 4a) and the resolution of interstitial and alveolar inflammatory infiltrates (Fig. 4b).

### Mechanisms of action—hMSC secretome

#### Epithelial wound healing

**Fig. 5:**
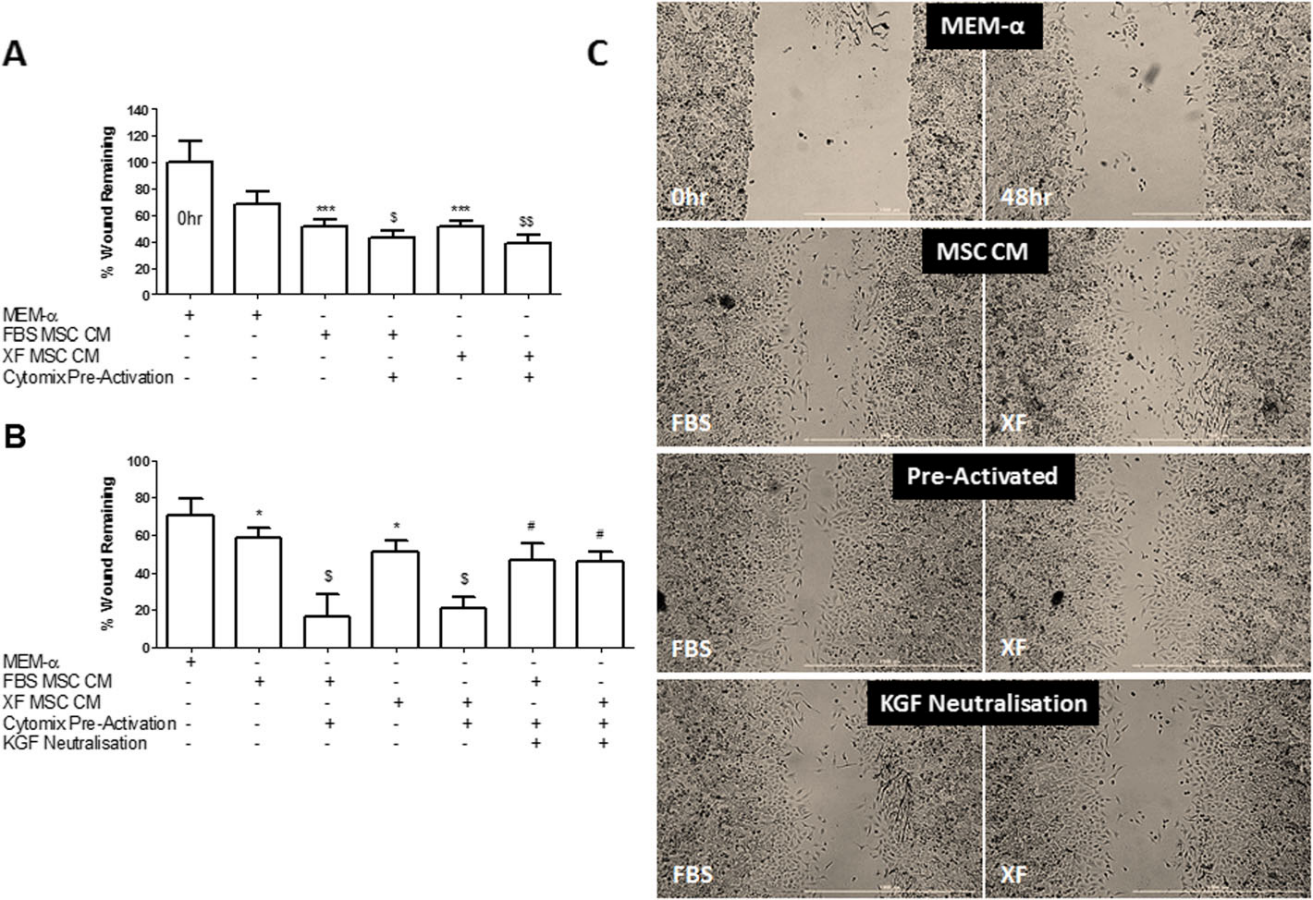
Pre-activation enhances the efficacy of hMSC CM in promoting pulmonary epithelial wound healing. FBS-hMSC CM and XF-hMSC CM comparably enhanced wound repair in comparison with the MEM-α control (**a**). Pre-activation of these MSCs further enhanced the ability of the CM to improve wound repair but this was attenuated by KGF neutralization (**b**). Representative images are provided (**c**). Note: * and *** = *P* < 0.05 and 0.001 versus MEM-α; and $ and $$ = *P* < 0.05 and 0.01, versus respective naive FBS or XF-hMSC CM; ^#^ = *P* < 0.05 versus respective pre-activated FBS or XF-hMSC CM. 0 h, *n* = 16; MEM-α control, *n* = 7–12; all other groups, *n* = 4–6

hMSC CM, whether from FBS- or XF-cultured cells, significantly improved pulmonary epithelial wound repair, an effect which was further enhanced with cytokine pre-activation (Fig. 5a). The effect of cytokine pre-activation was blocked by the addition of a KGF-neutralizing antibody (Fig. 5b, c).

## Discussion

In this paper, we present several important findings, each of translational significance. First, we demonstrate that the efficacy of MSCs was preserved when cultured in the xenogeneic material-free medium compared to the standard medium containing xenogeneic (bovine) products, an important advance. Second, we demonstrate that MSCs subjected to cryopreservation and thawing maintained efficacy compared to fresh MSCs in a relevant preclinical model of VILI. Third, we demonstrate the potential for cytokine pre-activation to enhance the efficacy of MSCs and specifically their potential to enhance the efficacy of cryopreserved xeno-free MSCs. Fourth, we illustrate the potential role of KGF in mediating some of the protective effects of these cryopreserved xeno-free MSCs in a relevant pulmonary epithelial repair model. Taken together, these findings address important translational concerns and significantly advances these cells towards clinical testing for patients with ARDS.

### Cryopreservation of hMSCs

Cryopreservation and storage of hMSCs would enable banking of hMSC batches that could then be thawed and administered in a timely fashion to patients with acute conditions such as ARDS. The alternative, namely provision of freshly cultured hMSC, would require that hMSCs are continuously available for harvest, in facilities adjacent to the clinical settings, which presents substantial logistical and economic challenges. Other challenges include the risk of increased cell batch variability and the potential for contamination of batches. However, several studies demonstrate that the cryopreservation and thawing processes may significantly reduce the efficacy of hMSCs [19, 40]. Our finding that cryopreserved XF-hMSCs can enhance the resolution of VILI when administered at therapeutically relevant time points in our rodent model supports recent findings in other models, including pneumonia-induced ARDS [41], and are important demonstrations of the feasibility of cryopreservation of hMSCs. This insight is further supported by the observation that the secretory profile of hMSCs in regard to IL-8 release, whether the hMSCs were pre-activated or not, remained the same pre- and post-cryopreservation.

### Xenogeneic-free hMSCs

Conventional culture and passage of MSCs requires the use of xenogeneic supplements to aid cell growth and replication, which includes the use of FBS that contains large amounts of growth factors [42]. However, the risks posed by the use of animal products in the culture medium of hMSCs intended ultimately for use in humans have been highlighted by the European Medicines Agency and US Food and Drug Administration [21]. Concerns include the risk of contamination or immunogenicity [22–25]. XF culture supplements would resolve these concerns. It has proven challenging to identify alternatives to FBS that maintain comparable growth conditions for MSCs and that do not adversely alter cell efficacy [42]. The recently patented XF supplement (WO2015121471 A1) has been demonstrated to preserve the differentiation, proliferation and low immunogenicity properties of MSCs [26]. Our finding that hMSCs cultured in this XF supplement retain the capacity to enhance the resolution of injury in our preclinical model of rodent VILI and maintain the secretome efficacy in attenuating pulmonary epithelial injury induced by cyclic stretch, and promoting wound healing in vitro, are important demonstrations of the therapeutic potential of XF-cultured hMSCs.

### hMSC secretome

MSCs release soluble anti-inflammatory and pro-repair molecules [43–45], a mechanism of action that has raised interest in the use of the MSC secretome as an alternative to MSC cell therapy. This study demonstrated that the secretome of XF-hMSCs protected the pulmonary alveolar epithelium from injurious cyclic stretch and also demonstrates that pre-activation with inflammatory cytokines enhanced secretome efficacy (Additional file 1: Figure S1 and Additional file 2: Figure S2). These findings were also true for the enhancement of wound healing. We further went on to show that KGF neutralization can negate the enhancement observed as a result of cytokine pre-activation. These outcomes extend findings from previous in vitro and in vivo studies showing that the MSC secretome resolved the inflammatory response and promoted repair and recovery post-VILI [13, 33] and thus strengthens the justification for the use of the secretome as a viable alternative therapy against VILI. However, it should be noted that the secretome may be less effective compared to hMSCs, especially in the early phases of the injury resolution process [46].

### Activation of hMSCs

MSCs are responsive to their microenvironment [44, 47] potentially providing a means of enhancing their efficacy via pre-activation strategies prior to their delivery. Recent studies have shown that MSCs pre-activated with inflammatory cytokines possess enhanced therapeutic properties [48]. Several other promising MSC activation strategies have been elucidated, including IL-10 overexpression [49] and interferon-γ priming [50]. Our studies advance our understanding of the therapeutic potential of priming by demonstrating that cytokine pre-activation can restore and/or enhance the function of cryopreserved XF-cultured hMSCs.

One aim in pre-activating cryopreserved MSCs with cytomix was to restore any therapeutic potency that might have been lost during cryopreservation. In regard to oxygenation, where cryopreserved MSCs only partially restored oxygenation, pre-activation did further enhance restoration of oxygenation. The second aim was to enhance MSC capacity to restore function post-VILI. In regard to lung compliance, where the effect of even fresh MSCs was limited, pre-activation enhanced both the cryopreserved and the fresh MSCs in restoring lung compliance.

Importantly, cytokine activation prior to cryopreservation enhanced the hMSC efficacy following storage and subsequent thawing and administration to the animals following VILI at a later and more clinically relevant time point. This provides an important finding with regard to other pre-activation or enhancement strategies for hMSC therapy applications, and it suggests such modifications are compatible with cryopreservation protocols.

### Limitations

There are a number of limitations to these studies. First, while we provide data to demonstrate that pre-activation of cryopreserved XF hMSCs enhances their ability to promote injury resolution following VILI and the ability of their secretome to reduce stretch injury and promote pulmonary epithelial wound repair, these studies were carried out in a rodent model and in vitro, and caution must be exercised in extrapolating to the clinical situation. However, we have used a clinically relevant injury resolution VILI model and utilized human MSCs. Thus, these findings strongly suggest that the therapeutic potential of cryopreserved XF-hMSCs for human ARDS may be enhanced by pre-activation measures. Second, we did not use a control non-stem cell group, such as a fibroblast group. In our previous experiments, rodent fibroblasts had no beneficial effect [35], while in experiments using human fibroblasts in rodents, some parameters indicative of injury were worsened [51]. Therefore, we believe that a fibroblast control is not justifiable for these studies. Our in vitro studies used A549 cells, an adenocarcinoma human alveolar basal cell line. These cells did have the advantage of being a widely used cell line, and they had an integrated NF-κB reporter. Finally, we do not provide data on a single overall mechanism of action of MSCs. Our studies to date [33, 35, 51, 52], and those of other groups [15, 53, 54], indicate that the effects of MSCs on, for example, innate and adaptive immunity, including antimicrobial effects, are varied, context dependent and not encapsulated by a single secreted mediator or group of mediators. Nonetheless, we show that the mechanisms of MSC therapy via their secretome as a whole is enhanced with pre-activation and is partly mediated through KGF.

## Conclusion

In conclusion, we demonstrate that hMSCs cultured in xenogeneic material-free medium enhance the recovery and resolution of VILI when administered at clinically relevant time points following the establishment of injury. These xenogeneic hMSCs were similarly effective following cryopreservation and thawing to fresh MSCs. Cytokine pre-activation further enhanced the efficacy of cryopreserved xeno-free MSCs. KGF may mediate some of the protective effects of these cryopreserved xeno-free MSCs. Taken together, these findings address important translational concerns and significantly advances cryopreserved xeno-free hMSCs towards clinical testing for patients with ARDS.

## Supplementary information

**Additional file 1: Figure S1.** hMSCs retain their secretary profile pre- and post-cryopreservation. IL-8 release by fresh naive hMSCs or fresh activated hMSCs is unaffected 24 h and 48 h post-thaw after cryopreservation. *n* = 3 for all groups.

**Additional file 2: Figure S2.** Pre-activated, cryopreserved, XF-hMSCs enhance the resolution of alveolar cell counts. Fresh, but not cryopreserved, XF-hMSCs decreased alveolar cell counts, while cytokine pre-activation restored the efficacy of cryopreserved hMSCs in reducing alveolar cell counts. ** and ***P < 0.01 and 0.001, respectively, versus PBS control; ^$$^*P* < 0.01 versus naive cryopreserved group. Sham, *n* = ; PBS control, *n* = 8; fresh, *n* = 8; fresh pre-activated, *n* = 7; cryopreserved, *n* = 5; cryopreserved pre-activated, *n* = 7.

## Data Availability

All data generated or analysed during this study are included in this published article.

## Abbreviations

ARDS: Acute respiratory distress syndrome
BAL: Bronchoalveolar lavage
BM-hMSCs: Bone marrow-derived hMSCs
CM: Conditioned medium
FBS: Foetal bovine serum
FGF: Fibroblast growth factor
hMSCs: Human mesenchymal stem/stromal cells
IFN: Interferon
IL: Interleukin
KGF: Keratinocyte growth factor
LDH: Lactate dehydrogenase
MEM-α: Alpha Minimum Essential Eagle Medium
PBS: Phosphate-buffered saline
TNF: Tumour necrosis factor
VILI: Ventilation-induced lung injury
XF: Xenogeneic-free
