# Supplementary figures and images for: Cytokine pre-activation of cryopreserved xenogeneic-free human mesenchymal stromal cells enhances resolution and repair following ventilator-induced lung injury potentially via a KGF-dependent mechanism
Source: Intensive Care Med Exp. 2020 Feb 5;8:8. doi: 10.1186/s40635-020-0295-5 (PMC7002627; doi:10.1186/s40635-020-0295-5)

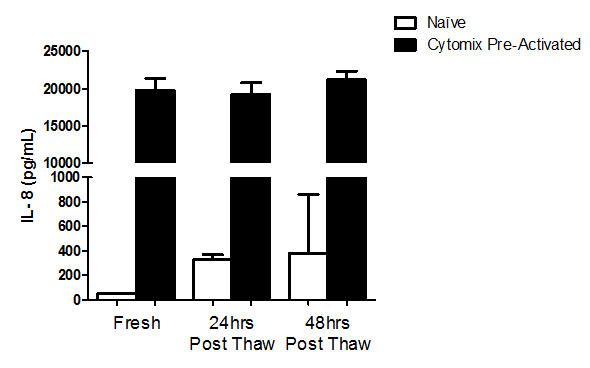

Supplement: Supplementary file 1 — Additional file 1: Figure S1. hMSCs retain their secretary profile pre- and post-cryopreservation. IL-8 release by fresh naive hMSCs or fresh activated hMSCs is unaffected 24 h and 48 h post-thaw after cryopreservation. n = 3 for all groups. [file 40635_2020_295_MOESM1_ESM.tif]

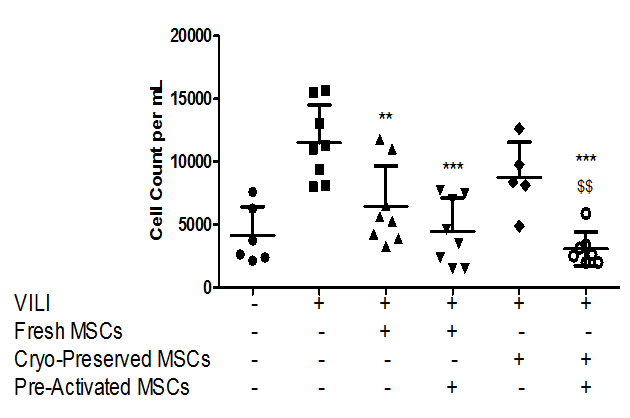

Supplement: Supplementary file 2 — Additional file 2: Figure S2. Pre-activated, cryopreserved, XF-hMSCs enhance the resolution of alveolar cell counts. Fresh, but not cryopreserved, XF-hMSCs decreased alveolar cell counts, while cytokine pre-activation restored the efficacy of cryopreserved hMSCs in reducing alveolar cell counts. ** and ***P < 0.01 and 0.001, respectively, versus PBS control; $$P < 0.01 versus naive cryopreserved group. Sham, n = ; PBS control, n = 8; fresh, n = 8; fresh pre-activated, n = 7; cryopreserved, n = 5; cryopreserved pre-activated, n = 7. [file 40635_2020_295_MOESM2_ESM.tif]
